# A Human, Organization, and Technology Perspective on Patients’ Experiences of a Chat-Based and Automated Medical History–Taking Service in Primary Health Care: Interview Study Among Primary Care Patients

**DOI:** 10.2196/29868

**Published:** 2021-10-18

**Authors:** Evalill Nilsson, Annette Sverker, Preben Bendtsen, Ann Catrine Eldh

**Affiliations:** 1 eHealth Institute Department of Medicine and Optometry Linnaeus University Kalmar Sweden; 2 Department of Activity and Health and Department of Health, Medicine and Caring Sciences Linköping University Linköping Sweden; 3 Department of Medical Specialists in Motala and Department of Health, Medicine and Caring Sciences Linköping University Linköping Sweden; 4 Department of Health, Medicine and Caring Sciences Linköping University Linköping Sweden; 5 Department of Public Health and Caring Sciences Uppsala University Uppsala Sweden

**Keywords:** digital encounter, digital healthcare, e-consultation, e-health, interview, patient perspective, primary healthcare, qualitative study, telemedicine, telehealth

## Abstract

**Background:**

The use of e-visits in health care is progressing rapidly worldwide. To date, studies on the advantages and disadvantages of e-consultations in the form of chat services for all inquiries in primary care have focused on the perspective of health care professionals (HCPs) rather than those of end users (patients).

**Objective:**

This study aims to explore patients’ experiences using a chat-based and automated medical history–taking service in regular, tax-based, not-for-profit primary care in Sweden.

**Methods:**

Overall, 25 individual interviews were conducted with patients in the catchment areas of 5 primary care centers (PCCs) in Sweden that tested a chat-based and automated medical history–taking service for all types of patient inquiries. The semistructured interviews were transcribed verbatim before content analysis using inductive and deductive strategies, the latter including an unconstrained matrix of human, organization, and technology perspectives.

**Results:**

The service provided an easily managed way for patients to make written contact with HCPs, which was considered beneficial for some patients and issues but less suitable for others (acute or more complex cases). The automated medical history–taking service was perceived as having potential but still derived from what HCPs need to know and how they address and communicate health and health care issues. Technical skills were not considered as necessary for a mobile phone chat as for handling a computer; however, patients still expressed concern for people with less digital literacy. The opportunity to take one’s time and reflect on one’s situation before answering questions from the HCPs was found to reduce stress and prevent errors, and patients speculated that it might be the same for the HCPs on the other end of the system. Patients appreciated the ability to have a conversation from almost anywhere, even from places not suitable for telephone calls. The asynchronicity of the chat service allowed the patients to take more control of the conversation and initiate a chat at any time at their own convenience; however, it could also lead to lengthy conversations where a single issue in the worst cases could take days to close. The opportunity to upload photographs made some visits to the PCC redundant, which would otherwise have been necessary if the ordinary telephone service had been used, saving patients both time and money.

**Conclusions:**

Patients generally had a positive attitude toward e-visits in primary care and were generally pleased with the prospects of the digital tool tested, somewhat more with the actual chat than with the automated history-taking system preceding the chat. Although patients expect their PCC to offer a range of different means of communication, the human, organization, and technology analysis revealed a need for more extensive (end) user experience design in the further development of the chat service.

## Introduction

### e-Visits in Swedish Primary Care

The digitalization of health care is now progressing rapidly, not least because of the current COVID-19 pandemic, when client-to-provider telemedicine (also known as digital, virtual, or e-consultations; digital encounters; or e-visits) may ensure access to health care at times of social distancing [1,2]. Such e-visits may include videoconferencing; emailing; text messaging via web portals; SMS text messaging; and, more recently, chat services, predominantly designed for mobile devices (especially mobile phones) with the intention of a quick reply.

Following a change in the legislation to increase access to primary care in Sweden, for-profit medical companies with web-based services only have offered chat services since 2014 for most patient inquiries in primary care for the entire population of Sweden, regardless of which primary care center (PCC) the patient actually belongs to. Although controversial from a reimbursement perspective (as an invoice will be sent to the patient’s regular PCC for each of such e-visits) and criticized for initiating certain treatments without a proper physical examination, these services have become increasingly popular for patients over the years. Subsequently, chat services are emerging also in regular tax-based, not-for-profit primary care, and contrary to the web-based medical services described earlier, the regular primary care offers the opportunity to convert chat conversations into clinical visits [3]. Despite the growing use of these chat services by patients, earlier interview studies have focused on the perspectives of health care professionals (HCPs) [4-7]. To our knowledge, before our study, no reports on patients’ experiences of chat services for all kinds of inquiries in regular primary care had been published, from neither Sweden nor elsewhere.

### Automated Medical History–Taking

e-Visit services often include automated medical history taking, as do the aforementioned chat services in Swedish primary care. Automated medical history taking can improve the quality of care and increase patient satisfaction; patients can document their medical history without any interference or time constraints, and HCPs can prepare for consultations in a time-efficient manner [8,9]. Earlier studies from primary care have suggested many benefits of using automated medical history taking, such as enabling triage prioritization and increasing diagnostic precision [10]. Potential risks include erroneous information, loss of nonverbal cues, and low digital literacy. As long as the questions are carefully designed to describe their health status accurately, patients using automated medical history taking in emergency care have reported that it helps them to organize their thoughts and thus enables better dialog with their physician [11].

### Human, Organization, and Technology Perspectives on e-Visits

The rapid introduction of new and innovative digital communication tools in health care, often without taking enough time to consider implementation strategies from a theoretical perspective, has highlighted the need for thorough evaluations. Studying the implementation of e-visits from the three perspectives of human, organization, and technology (HOT) in parallel and given equal attention can render a more holistic evaluation [12-15]. Human factors include aspects such as user satisfaction; organizational factors cover aspects such as structure; and technological factors include aspects such as information and system quality. The more these aspects fit with each other, the greater the potential. The greatest risks are found in the intersections and interactions among them, and therefore, it is important for successful implementation to identify and rectify any gaps.

The aim of this study is to explore patients’ experiences of using a chat-based and automated medical history–taking service in Swedish, regular, tax-based, not-for-profit primary care.

## Methods

### Design

An explorative design formed this study, with data collected through semistructured individual interviews and analyzed using content analysis.

### Setting

Health care in Sweden is regionalized and managed by 21 independent county councils. In the county of Östergötland in southeastern Sweden, a chat-based service (provided by a commercial company) within primary care was tested by 5 PCCs for about a year, starting from April and May 2019. These PCCs were representative of the region, and Sweden, including both urban and rural areas. Normally, patients who need to consult their PCC for various health-related requests would contact their PCC through a telephone triage system, where they register their telephone number and are assigned a specific point in time later in the day when they will be contacted by a nurse at the PCC for triage, that is, Swedish patients are not accustomed to being put in touch directly with their personal physician, nurse, or physiotherapist, when contacting their PCC. When all available time slots for talking to the triage nurse are taken for a particular day, patient callings are advised automatically by the system to try again the following day.

During the test period, adult patients calling their PCC between 8 AM and 3 PM, Monday to Friday (weekdays), were offered the use of a digital chat-based communication system as an alternative to the telephone triage. A voice message specified the alternative, offering the choice of receiving a link to the digital service via a text message on their mobile phone, along with a guaranteed response in the chat from the PCC within 2 hours. It was also possible to access the service through the websites of the PCCs. To log in to the system, the patient used a common secure personal identification system. Before the actual chat, an automated medical history–taking service was offered, where the patient responded to a battery of automatically generated questions about their chief complaint and current health status. The questions were rule-based, although response adaptive, and included both fixed and free response alternatives.

### Sample

Patients logging in to the chat service in September to November 2019 were asked if they were willing to participate in the evaluation study. If so, they ticked a box giving the researchers permission to contact them with more information about the study. During this period, nearly 600 patients ticked the box and 25 of them were selected (5 from each PCC) in stratified purposeful sampling, to ensure participation of both men and women of all ages. In addition, age- and sex-matching reserves were selected if patients had changed their minds or otherwise were unable to participate in the study. In total, 35 patients had to be invited as 10 declined for various or unknown reasons. The demographics of the participants are presented in Table 1.

**Table 1:** Demographics of the participants (N=25).

Demographics			Total (N=25)		Women (n=13)		Men (n=12)
Age (years), mean (SD; range)			51 (19.0; 21-81)		48 (19.3; 21-81)		53 (18.4; 22-76)
Living in urban areas, n (%)			19 (76)		9 (69)		10 (83)
**Manage your own electronic devices,^a^ n (%)**							
	Always or almost always	23 (92)		11 (84)		12 (100)	
	Most of the time	1 (4)		1 (8)		0 (0)	
	Some of the time	1 (4)		1 (8)		0 (0)	

^a^Nobody chose the response alternative *never or almost never.*

### Procedure

#### Data Collection

The selected patients were contacted via the mobile phone number registered while using the chat service. Information about the study was given orally and also sent by mail or email to those who agreed to participate in the study. A suitable time for the telephone interview was agreed upon, according to the availability of the patient.

The interviews were conducted by 2 of the researchers in the team: author AS conducted 12 interviews and a trained research associate (Catharina Linderoth) conducted the other 13 (as further noted in *Acknowledgments*), supported by an agreed semistructured interview guide compiled for this study. The guide comprised three areas: (1) digital communication (in general), (2) experience of the digital tool in question, and (3) digital patient-professional relationship. Probes were available but were used only if the respondents did not naturally expand on the subjects. Four final demographic questions were asked: (1) age, (2) sex, (3) whether the respondents considered themselves living in a rural or urban area, and (4) how confident the respondent was in managing problems with their electronic devices (Table 1). The interview guide was tested and validated in a pilot study at the beginning of the test period (April-May 2019), indicating that it was comprehensive and corresponded to the study purpose. Before each interview began, the participant received a recap of the study information before the participant’s informed consent, which was recorded. All interview recordings were transcribed verbatim by a skilled secretarial service. The interviews lasted between 8 and 31 minutes, with a mean of 21 (SD 5) minutes. The transcripts rendered 153 pages of one-and-a-half-spaced text. No data were reported back to the participants or PCCs.

#### Data Analysis

All transcripts were read separately by all authors in an inductive manner and everyone then presented, in writing, their individual general understanding of the data set, per interview and as a whole. A subsequent team discussion informed a common understanding of tentative categories. To advance the structured analysis, the elements of HOT were considered by the team, generating an unconstrained matrix, as described by Elo and Kyngäs [16]. To conclude, the relationships among the elements were mastered and quotations illuminating the findings were identified across the data set [17].

## Results

### Overview

**Figure 1:**
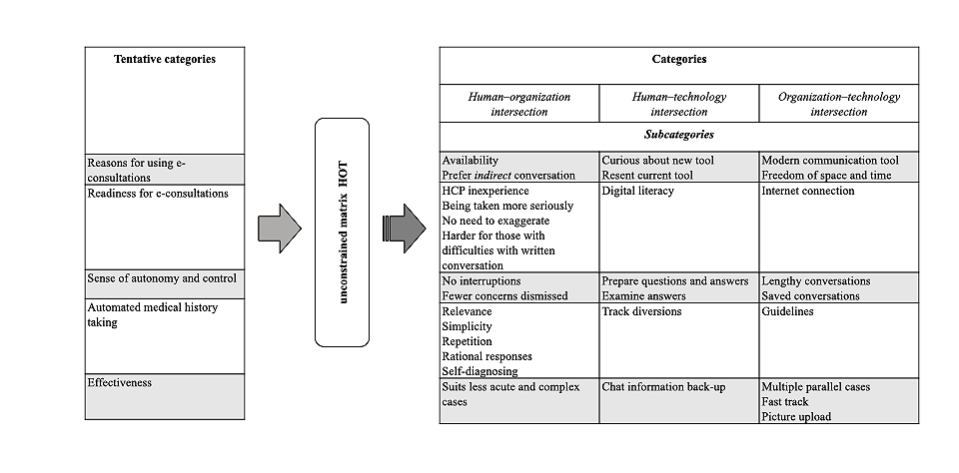
The forming of categories by means of the unconstrained matrix: tentative categories analyzed from an HOT perspective and subcategories informing the intersection among the three elements (human, organization, and technology). HCP: health care professional; HOT: human, organization, and technology.

Patients’ experiences of the chat-based and automated medical history–taking service signify intersections among HOT aspects. An overview of the categories identified in correspondence with all three HOT elements is presented in Figure 1, along with the intersections among the three elements, which are described in more detail later.

### The Human-Organization Intersection

Patients trying out this new digital communication tool thought it would be a quicker way to get in contact with their PCC. A more specific reason was the preference for written communication, for example, because talking to other people induces anxiety, especially if the subject is health related, the issue is sensitive or embarrassing to raise face-to-face, or simply because it suits them better than oral communication:

When I need to get in contact with healthcare it induces anxiety, which makes it very difficult for me to pick up the phone and make the call. This makes me postpone it for far too long and therefore this chat service has made things much easier for me.

Interview #13

However, the patients raised concerns for other patients with reading and writing difficulties or vision impairment. Furthermore, digital chatting was not considered an optimal way to communicate regarding emergencies and severe illnesses or when a deeper and more intimate conversation was required.

Regarding the automated medical history–taking service, patients found the questions fairly easy to understand, but they were uncertain about the accuracy of their own responses, wondering which information would be vital and which would be redundant. In the absence of clear guidelines, patients began diagnosing themselves by trying to answer the questions. Furthermore, from their point of view, patients were both lacking relevant and receiving irrelevant and repetitive questions, some of which pertained to issues that the patients felt sure were already documented in their electronic health record (EHR):

When you talk [on the phone] to the nurse or caregiver they will ask follow-up questions, so that you understand better what it’s all about. To express yourself verbally and not have the right knowledge behind it could easily become difficult.

Interview #04

In the subsequent chat, patients were never interrupted, and their concerns were less easily dismissed than in telephone calls; rather, they sensed that they were taken more seriously in the chat. Therefore, they did not feel the need to exaggerate their symptoms to get an appointment. The medical assessment was mainly regarded as fair and the advice as constructive. However, as some personnel seemed to be more skilled than others, the patients made suggestions regarding further training in written communication and digital chatting for HCPs:

In the chat you can say all that you want to say without anyone hanging up or interrupting or making something up just to dismiss you. So, all information will be put forward and they will read it and have to take it more seriously than over the phone.

Interview #06

Overall, data show that, regarding the human-organization interaction, patients expected the organization to be more prepared for chatting than turned out to be the case.

### The Human-Technology Intersection

The technical standard of digital services is important to patients. As the patients expressed some negativity regarding the current telephone triage system, there was an openness to new tools. Common curiosity with new technical solutions was a reason for trying out the new service. Although all participants considered themselves able to manage most everyday problems with their electronic devices, technical skills were not considered necessary for the chat in the same way as for handling, for example, one’s computer. Yet, patients conveyed concerns for people with less digital literacy, such as equity concerns about the service not aiding older people properly, as older people as a group are the least digitally literate group in society.

Although patients appreciated the potential benefits of an automated history-taking system, they expressed a sense of unease in answering the questions in certain ways, leading to undesired consequences, such as selecting a response alternative that guided them away from the line of questioning that they felt was relevant, with no opportunity to retract. The *back-up*, that is the possibility in the subsequent chat for correcting any mistakes from the automated history-taking procedure, was considered reassuring but ineffective, duplicating efforts.

The opportunity for patients to take their time and reflect on the situation before answering questions from HCPs was stress reducing, error preventing, and facilitated follow-ups. Besides being less stressful for themselves, patients contemplated how chatting would be less stressful also for the HCPs for the same reasons:

Writing everything down means I can check if I have forgotten anything. I can list things and add text before I send it, but when you talk to people you might forget things, important things, and when we are done talking, I remember, oh, I should have mentioned this and that!

Interview #09

### The Organization-Technology Intersection

As web-based chatting in general nowadays is a common service in Sweden, in commercial and public sectors, patients suggested that people have become accustomed to this kind of customer-centric digital service. The patients anticipated that their usual HCPs would offer similar services. The chat permitted patients to have a conversation from almost anywhere, even from places not suitable for telephone calls, such as at work or on a bus. However, although most other chat services in society are synchronous, the chat in this study was asynchronous, which was disappointing to some patients. Although asynchronicity allows patients to take more control over the conversation and initiate a chat at any time at their own convenience, it could also lead to lengthy conversations where a single concern in the worst case could take days to close. This meant that health care personnel at times had been replaced by others in the chat, leaving the patient with a sense of having to start all over again. However, at the same time, patients reported that an asynchronous chat service supposedly provided efficiency, with personnel being able to handle several cases simultaneously. A chat service is expected to be a faster route of communication than, for example, a telephone service; however, this was not always the case, which patients attributed partly to the asynchronous structure of the chat and partly to staff’s inexperience with the system, anticipating that the latter would change and improve over time:

I think the difficulty lies in the inactivity in the chat. Because when they write a question and I respond and it takes them hours to answer they will have lost the flow, because they have been doing many other things in-between. It felt like they neither had the energy nor the time to go back in the text flow, which meant they had to start all over again, to some extent.

Interview #19

The opportunity to upload photographs made some visits to the PCC redundant that would otherwise have been necessary if a telephone service had been used, saving both time and money of patients. However, an adequate internet connection is required, not least when uploading photographs, which was pointed out as an obstacle:

To be able to upload pictures of, for example, wounds and eczemas and stuff like that, that makes it [the chat service] nothing but positive.

Interview #16

The fact that technology allows for the (whole) conversation to be saved word for word, also in the EHR, for future reference was described as a new aspect to take into account when expressing oneself in the chat contacts; the patients were used to the HCPs summarizing the conversation in the EHR by extracting only vital aspects and using concise and professional language. Furthermore, improvements such as a fast track for simple cases and guidelines in the system for answering the questions were found to be desirable:

It is possible to follow up from both sides, they can see what I have written and they can see what others that work there have answered as well. The follow-up is better than with telephone conversations.

Interview #18

In summary, patients were predominantly positive about the chat service and felt it added value, even emotionally:

I felt welcomed, and that is not always the case.

Interview #23

## Discussion

### Principal Findings

The results from this study indicate that patients would welcome the chat service becoming permanent, as one of the several ways of contacting their PCC. However, there are several aspects from the HOT perspective that must be considered before implementation across primary care.

A health care organization will have to balance the desire to provide access and simplicity for patients with the obligation to provide healthy and safe working conditions for staff and uphold data privacy, security, and high-quality care (*human-organization*). Compared with the earlier study from our research group regarding the perspectives of HCPs, patients in this study seemed to be somewhat more satisfied with the chat service than the HCPs were [4]. For example, although anxious to offer patients ample availability, HCPs reported that patients used the chat service to gain quicker access to health care regardless of the degree of urgency of their health problems and sometimes initiated contact for the same complaint through several channels in parallel, which may add to the workload of primary care. As the chat was asynchronous, it was not always the fast route that patients anticipated, and perhaps the service should be renamed and the term *chat* should be reserved for synchronous services.

Both patients in this study and HCPs in the earlier study [4] regarded the chat as being more on the patients’ terms than traditional face-to-face or telephone communication, although patients, by comparison, seemed to find that as more positive than did the HCPs. Although no earlier interview studies were found with patients regarding modern and fast, primarily mobile phone based, chat services for all inquiries in primary care, there are several studies (both interview and questionnaire studies) regarding patients’ views on other text messaging services in primary care, for inquiries in general, such as in this study, and for the self-management of chronic diseases or health-related interventions, where the patient-professional contact has already been established. The results of these studies are consistent with the findings of this study regarding patients’ views on the appropriateness and availability of e-visits and the pros and cons of written communication [18-23].

How patients are received by HCPs is the most common grievance when assessing health care from a patient safety perspective [24]. Although chatting may diminish problems such as interruptions and not getting enough time to express yourself, new problems seem to emerge instead, such as HCPs inexperience with professional chat communication about health and health care issues. Interestingly, regarding the chat service in this study, this issue was only raised by patients and not by the HCPs themselves [4]. One might expect patients’ opinions to change when the service has settled after having been operational for some time; however, in fact, patients’ views in this study were unexpectedly similar to those in interviews conducted during the pilot study in May 2019 (data not yet published). Further studies are needed to examine if patient safety is affected by this line of communication and if training of HCPs in written communication and chatting would be beneficial [25,26].

The automated medical history–taking service is meant to provide health care personnel decision support and faster triage and management. This requires that the questions are relevant and easy both to understand and respond to. Verbal anamnesis, or medical history taking, is usually documented by HCPs asking the patient questions and follow-up questions, guiding the story, and immediately correcting mutual errors and misunderstandings. The automated medical history–taking service in this study seemed to be instigated by the needs of the organization rather than by the needs of the patient. Naturally, the choice of questions must have a medical basis, but patients would have much to add, for example, in which cases questions are perceived as hard to respond to, irrelevant, or inexplicably repetitive and when questions of relevance to them seem to be missing. The health care organization cannot simply assume that questions that are obvious to trained professionals will be automatically understood by patients and should not underestimate the importance of patients having the opportunity to convey all their concerns as they see fit. Patients’ suggestions about a more pronounced user experience design approach when developing automated medical history–taking systems should be given consideration (*human-technology)* [18].

An adequate internet connection was considered an important prerequisite for using the new digital communication tool, but it is a societal rather than merely a health care concern. On the other hand, patient privacy and data security are very much health care concerns (*technology-organization*) [27]. Contrary to early studies from the beginning of the 21st century about patient-provider contacts via email in primary care, for example, where patients raised concerns about security and lost messages [28], patients in this study did not raise any cybersecurity or legal issues during the interviews. This may be interpreted as a solid trust in the system or a lack of awareness of the risks of digital communication, either in general or at the time of the interview. Trust has been shown to have a positive correlation with the intention to use e-consultation services [29]; if so, respondents lacking trust most likely would not consider trying the service in the first place and, therefore, were not available for this study with its current design.

Earlier studies have shown that organizational elements are crucial for the successful implementation of health information technology [30]. In this case, the chat service was trialed following only a limited training session for the staff engaged (organized by the commercial company providing the digital tool and only regarding the technical and administrative aspects of the tool, not regarding how to create a therapeutic alliance with patients using digital tools) [4] and very limited information to the patients. Furthermore, aspects such as cost-effectiveness will impact the implementation process. For example, the intention was to staff the service so that the person responsible for the chat could concentrate on that task and not have to alternate with other tasks. However, because of limited resources, this was not possible at all PCCs, as the influx of cases was not enough to warrant the higher cost of assigning a nurse solely to the chat service for all workdays (8 hours a day) [4]. Thus, the stakeholder’s perspective was, to a certain extent, missing in both the creation and implementation of the chat service, and therefore evaluation from this perspective is even more important. Findings from this study indicate that patients provide perspectives of interest to further planning and implementation, confirming that the organization, technology, and human and social aspects need to be considered throughout the implementation of health technology innovations [12,30].

### Methodological Discussion

Engaging patients in quality improvement is a suggested standard procedure, and the end user perspective is vital for any health care development [31]. However, a limitation of this study is that the participants may constitute a sample of people more used to computers and mobile electronic devices than others; having a mobile phone, tablet, or computer was a technical prerequisite for using the chat service. To explore the technical interface in a wider context, respondents should represent those less used to electronic devices as well. However, digital illiteracy may not be the major reason for not choosing to engage in a digital encounter; nonusers often report simply preferring to meet and speak with their health care providers [28,32]. Nevertheless, the novelty of this study is its inclusion of a variety of people with firsthand experience as patients using a digital chat with their primary care provider regarding all possible inquiries; thus, it may be of interest for similar contexts and further investigations of tools enabling digital communication [33].

### Conclusions

Patients in general had a positive attitude toward e-visits in primary care and were generally pleased with the prospects of the digital tool, somewhat more with the chat service than with the automated history-taking system preceding the chat. Although patients expect their PCC to offer a range of different means of communication, the HOT analysis revealed a need for more extensive (end) user experience design in the further development of the PCC chat service.

## Abbreviations

EHR: electronic health record
HCP: health care professional
HOT: human, organization, and technology
PCC: primary care center
